# Goitre

**Published:** 1907-03

**Authors:** 


### GOITRE.

---

C. H. Mayo (Journal A. M. A., January 26, 1907) observes that the general impression to be obtained from a review of the surgical literature of America would be that diseases of the thyreoid gland are greatly on the increase. This is probably not the case, but the public has learned that operations on goitre are not as fatal as were supposed from the results obtained when operations were made as a last resort on patients suffering from this disease and in a moribund state. The fact is that the mortality attending the operation (excluding cancer and advanced cases of exophthalmic goitre) compares very favorably with other major surgery; and, in the hands of those experienced, with much of the so-called minor surgery. Mayo describes the operative as follows: The choice of an anæsthetic is more often determined by the idiosyncrasy of the operator than the necessity of the case. There were thirteen operations made with cocaine infiltration. Of these three were completed with chloroform. While there are occasional cases in which local anæsthesia may be necessary, the author has noticed but little difference in the shock or general condition of the cases from the anæsthetic employed. He now uses ether anæsthesia, preceded twenty minutes by hypodermic of 1-6 gr. of morphine and 1-120 gr. atropine. The table is placed in an elevated slanting position with the head up (reverse Trendelenburg). The tumor is rendered prominent by a roll beneath the neck if it does not interfere with respiration. He prefers the collar or transvese incision. In tumors of medium size this crosses the centre of the tumor, in larger tumors it crosses the upper third and the lower flap is split vertically to the sternum if necessary. The incision is through skin and platysma muscle, the flap being raised to expose freely the muscles covering the gland, namely, the sternohyoid and thyreoid, the inner portions of the sternomastoid and omohyoid. In medium sized growths muscle separation will permit of the delivery of the tumor. After the removal of the gland, the severed muscles are carefully united by suture. The upper section also permits of early ligation of the superior thyreoid artery which is the key to the situation. The more firm and rounded tumors, with a strong capsule, can be readily enucleated after incising the capsule and penetrating the gland tissue to the cyst. He usually loosens the gland from the outside of the capsule first. On viewing the tumor, the true capsule has the lustre and appearance of peritonæum. If one is not sure, incise between the vessels. Should hæmorrhage be severe, lifting up the tumor by its capsule will reduce the bleeding until sutures can be placed for its control. In parenchymatous and colloid tumors he makes extirpation of one lobe and the isthmus. After exposing it the upper pole is elevated and the superior thyreoid artery is cut between the ligatures. The lateral veins are clamped and cut, the lower pole is elevated into the wound, incision is made along the outer posterior border, and the capsule is brushed off wit hgauze to the median line. The isthmus is clamped and closed by suture. If the enucleation method for adenoma and cysts leaves a badly torn lobe it can be removed. The results the author had were very satisfactory.

